# Heterogeneity of Mitochondria and Mitochondrial Function within Cells as Another Level of Mitochondrial Complexity

**DOI:** 10.3390/ijms10041911

**Published:** 2009-04-24

**Authors:** Andrey V Kuznetsov, Raimund Margreiter

**Affiliations:** 1 Daniel Swarovski Research Laboratory, Center for Operative Medicine, Department of Visceral, Transplant and Thoracic Surgery, Innsbruck Medical University (IMU), Innrain 66, A-6020 Innsbruck, Austria; 2 Center for Operative Medicine, Department of Visceral, Transplant and Thoracic Surgery, Innsbruck Medical University (IMU), Anichstrasse 35, A-6020 Innsbruck, Austria

**Keywords:** Mitochondrial integration, mitochondrial morphology, mitochondrial membrane potential, mitochondrial specializations, subpopulations

## Abstract

Beyond their fundamental role in energy metabolism, mitochondria perform a great variety of other important cellular functions. However, the interplay among these various roles of mitochondria is still poorly understood, and the underlying mechanisms can be related to system level properties. Importantly, mitochondria localized in different regions of a cell may display different morphology, dissimilar biochemical properties, or may differently interact with other intracellular structures. Recent advances in live imaging techniques have also revealed a functional heterogeneity of mitochondria with respect to mitochondrial redox state, membrane potential, respiratory activity, uncoupling proteins, mitochondrial ROS and calcium. An important and still unresolved question is how the heterogeneity of mitochondrial function and the regional specializations of mitochondria are mechanistically realized in the cell and to what extent this could be dependent on environmental aspects. Distinct mitochondrial subsets may also exhibit different responses to substrates and inhibitors and may vary in their sensitivity to pathology, resistance to apoptosis, oxidative stress, thus also demonstrating heterogeneous behavior. All these observations strongly suggest that the intracellular position, organization and the specific surroundings of mitochondria within the cell define their functional features, while also implying that different mitochondrial subpopulations, clusters or even single mitochondrion may execute diverse processes in a cell. The heterogeneity of mitochondrial function demonstrates an additional level of mitochondrial complexity and is a new, challenging area in mitochondrial research that potentially leads to the integration of mitochondrial bioenergetics and cell physiology with various physiological and pathophysiological implications.

## 1. Introduction

Mitochondrial imaging was used intensively to analyze important functional and morphological characteristics of mitochondria in living cells, as well as their intracellular distribution and arrangement [1–6]. Recently developed methods also include: fluorescence resonance energy transfer (FRET) for imaging protein-protein interactions and conformational changes; recording the image data in three spatial dimensions over time (i.e. 4D imaging), as well as 4Pi and STED microscopy [7,8]. Various imaging techniques permit both spatial and temporal study of mitochondrial morphology and function. Confocal imaging of mitochondria in living cells of several types revealed that mitochondria can be either clustered or arranged in a highly organized, tissue-specific manner [9]. Several mitochondriaspecific markers or fluorescent proteins specifically targeted at mitochondria are now widely used in combination with high-resolution imaging techniques. Furthermore, confocal fluorescence microscopy distinguishes fluorescence originated from different mitochondrial subpopulations, small clusters or even from single mitochondrion in distinct cell regions, thus permitting quantitative analysis of the region-specific function of these organelles [3,5,6,10]. These imaging approaches are also most direct means of visualizing the intracellular heterogeneity of mitochondria and mitochondrial function. Using confocal fluorescent microscopy, mitochondrial heterogeneity has been demonstrated in various cell types [3,5,6,11–14]. Functional heterogeneity of mitochondrial membrane potentials and calcium has been reported for a wide spectrum of cells, including hepatocytes, HUVEC, astrocytes, HL-1 cells, fibroblasts, muscles and various cultured human carcinoma cells [3,12]. Also mitochondrial heterogeneity can be seen from their different redox states, respiratory activities, different levels of uncoupling proteins and mitochondrial ROS [5,6,10–14].

In some cells like neurons, a highly dynamic organization of mitochondrial networks can be achieved by these organelles moving along the cytoskeleton and attaching to the cytoskeleton using specific motor and connector proteins [15]. Alternatively, in adult cardiomyocytes mitochondria are organized in a “lattice” of parallel rows surrounding the contractile myofilaments and are rather fixed in their positions [16], forming a regular (crystal-like) arrangement [9]. This type of organization provides a bioenergetic basis for contraction and multiple interactions of mitochondria with other intracellular systems like the endoplasmic reticulum and the cytoskeleton (termed ICEUs), recruiting various cytoskeletal proteins [17–22]. Moreover, the mitochondrial interactions with cytoskeletal proteins (like desmin, vimentin, tubulin or plectin) can effectively modulate mitochondrial respiration [23–26]. Numerous recent studies show that the precise organization of mitochondria and numerous interactions between these organelles and other cellular structures and systems play a fundamental role in mitochondria regulation *in vivo* in living cells [2,20–22,24,27]. All these data support the concept of the functioning of complex systems for energy transfer, including metabolic compartmentalization and coupling between enzymes and organelles, in order to achieve specific functions for particular cellular requirements. The challenge in bioenergetics, therefore, is to explain the roles of mitochondrial organization in the cell and multiple interactions of mitochondria with other intracellular systems.

## 2. Overview of Existing Results and Their Discussion

### 2.1. Multiple functions of mitochondria in the cell

**Figure 1.**
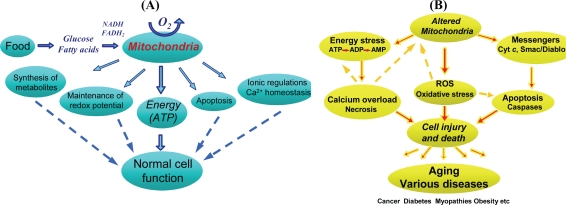
Key roles of mitochondria in normal cell function (A) and injury (B).

Imbalance between energy production and energy demand and a disturbance in energy transfer networks play an important role in various pathologies, and this complex problem is currently a cornerstone of intense research being conducted in several sub-specialties of physiology. Most of the energy (on the form of ATP) is provided in the cell by mitochondria, placing these organelles center stage in many aspects of cell biology and medicine. Definitely, mitochondria are now recognized not only as the main intracellular source of energy in the form of ATP needed for normal cell function and viability, but also as a major controller in many cellular pathways, performing a great variety of other important cell functions (Figure 1A) [28].

These organelles regulate the cellular redox state and play very important roles in ionic regulations (in particular in calcium homeostasis) and in apoptosis [29,30] and can be considered an integral part of multiple cellular signaling and a mediator of cell communication and survival [31–35]. Mitochondria participate in Ca^2+^ signaling as a result of their close apposition to Ca^2+^ release (endoplasmic reticulum, ER [19]) and Ca^2+^ entry sites (plasma membrane), where microdomains with high local Ca^2+^ concentrations are formed. Moreover, mitochondria are directly involved in pathophysiological mechanisms of ischemia reperfusion injury, oxidative stress, preconditioning, inherited diseases, toxicological injury, and side-effects of pharmacological treatments. Damaged mitochondria cause organ injury also by several mechanisms, including the diminished cellular energy status (energy stress), production of reactive oxygen species (oxidative stress), disturbance of ionic balance, cytochrome *c* release and induction of apoptosis (Figure 1B). Although many mechanisms involved in mitochondrial function and regulation have been studied quite extensively, the interplay among the different roles of mitochondria is still poorly understood.

### 2.2. Heterogeneity of mitochondrial morphology and organization

Broad variations in mitochondrial shape and morphology can be observed in various cells, but also within one cell, including small spheres or short rod-like shapes, long filamentous spaghetti-like mitochondria, together with complex, branched mitochondrial networks found for example in HL-1 cells, human pancreatic cells, etc. [6,36]. In some cell types mitochondria exist as single and randomly dispersed organelles [3,9,13]. In other cells, mitochondria may also exist as dynamic networks that often change shape and subcellular distribution [48]. In these cells, fusion causes formation of mitochondrial reticulum, which may play an important role in cell physiology. Several other cell types like adult cardiomyocytes show functionally and structurally distinct mitochondria [3,9,12,46], which is also very important for the specific functions of these cells [20,21]. Significantly, mitochondrial dynamics (fission/fusion), which controls morphology and organization of these organelles can be important also for their functioning and metabolism regulation (i.e. mitochondrial shape might have crucial consequences for mitochondrial function) [28,58,61]. It has been shown that mitochondrial function is reduced in cells with deficiency of proteins responsible for mitochondrial fusion [61]. These cells demonstrate also cellular defects, such as reduced growth rates, reduced cellular respiration, and the presence of highly heterogeneous mitochondria, which may suggest adaptation to the reduction of mitochondrial functionality.

**Figure 2.**
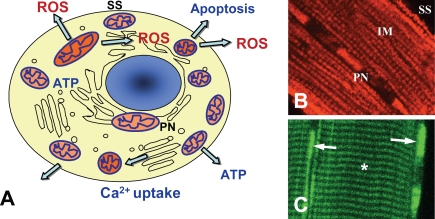
(A) Mitochondria-specific function may be dependent on the particular cellspecific region and surrounding. (B) Subsarcolemmal (SS); intermyofibrillar (IM) and perinuclear (PN) mitochondrial subpopulations in rat cardiomyocyte visualized by TMRM (0.1 μM). (C) Subsarcolemmal (arrows) and intermyofibrillar mitochondria (asterisk) in rat skeletal muscle (m. soleus) show very different intensity of flavoprotein autofluorescence, demonstrating different redox state of these subpopulations.

Mitochondrial clustering has been reported as one specific organization in various cell types that may also be associated with specific cellular demands. For instance, mitochondrial clusters surrounding the nuclei in cardiomyocytes (Figure 2B) may serve to drive mitochondrial metabolism to generate ATP close to the nucleus. Such a clustering may play an important physiological role in the mechanisms for nuclear import [14,37]. In general, the diversity of mitochondrial shapes and intracellular arrangements can be explained by their highly specialized cellular functions in the cells with different energy demands and preferences in the type of mitochondrial substrates, but also by the differences in their physiological state (energy and redox states, membrane potentials and calcium levels).

It is well known that the cytoskeletal proteins are absolutely crucial for mitochondrial morphology, motility, intracellular traffic and mitosis [17]. Mitochondria are associated with the three major cytoskeletal structures: microfilaments, microtubules and intermediate filaments [38,39]. In many cells and tissues, mitochondria typically display a subcellular distribution corresponding to that of the cytoskeletal network and many chemical agents that depolymerize microtubules significantly modify intracellular distribution of mitochondria [38,40]. However, mitochondrial interactions with the cytoskeleton are shown to be very important, not only for control of their morphology and organization, but also for their functioning. A growing body of evidence suggests that cytoskeletal network and specific cytoskeleton-associated proteins may interact with mitochondria to regulate mitochondrial respiratory function by controlling the permeability of the mitochondrial outer membrane to ADP. For example, experiments performed with desmin-null cardiac cells have indicated that the respiratory function of mitochondria *in vivo* can be significantly changed in these cells [18,24]. Remarkably, a decrease in the maximal rate of ADP-stimulated state 3 respiration and in the apparent Km for ADP in desmin-null cardiac and soleus muscles was reported, although these two major characteristics were not changed in another type of desmin-null muscle, in the glycolytic m. gastrocnemius. Moreover, ultrastructural analysis of desmin-null cardiomyocytes has demonstrated widespread proliferation of mitochondria that was increased after work overload. Very recent evidence shows that another cytoskeletal protein tubulin can also control the permeability of the mitochondrial outer membrane [25]. The addition of dimeric tubulin induces reversible closure of the reconstituted voltage-dependent anion channel (VDAC). Furthermore, in the model of isolated (*in vitro*) mitochondria tubulin can restore the low permeability of the outer membrane [25], increasing apparent Km for ADP to the value of *in situ* mitochondria (found in permeabilized fibers or cells [26,41]). Recent findings also suggest that vimentin and plectin-1b isoform are critical for the association between the mitochondria and the cytoskeleton in some cells [42,43], contributing to the maintenance of mitochondrial morphology and intracellular organization, and potentially playing an important role also in the regulation of mitochondrial function. It has been shown that in oxidative muscles the mitochondria are organized into functional complexes with myofibrils and sarcoplasmic reticulum, creating intracellular energetic units (ICEUs). Within these complexes, the energy crosstalk involves the facilitated diffusion of ADP and metabolic channeling in the local energy transfer networks, including the creatine kinase and adenylate kinase systems, and thus representing the basic pattern of organization of energy metabolism in oxidative cells [20–22,44].

### 2.3. The connectivity of mitochondria

The connectivity of mitochondria in living cells has been addressed in several studies using a range of confocal imaging techniques [3,12,16,19,45,46]. In some cells, mitochondria exist as single and randomly dispersed organelles. In other cells, mitochondria may also exist as dynamic networks that often change shape and subcellular distribution. Application of the imaging approach has revealed electrical connectivity of a mitochondrial network in cells like human skin fibroblasts, COS-7 cells [47], and neonatal rat or HL-1 cardiac myocytes [16,48]. Mitochondrial imaging and the fluorescence recovery after photobleaching (FRAP) technique have demonstrated the existence of an interconnected branched mitochondrial network also in HeLa cells [19]. Alternatively, many distinct mitochondria in HeLa and various other cells can be consecutively depolarized, indicating existence of electrically nonconnected mitochondria [3,12,45]. In these experiments, asynchronous depolarization events induced by laser irradiation clearly indicate the positions of the electrically isolated mitochondria and do not indicate electrical relationships between these organelles [45]. A similar phenomenon of the electrical discontinuity of mitochondria was found in adult cardiomyocytes [16,46]. It has been shown that laser irradiation resulted in the collapse of the membrane potential of distinct, separate mitochondria due to local ROS production and permeability transition [6,16,46,49–51]. This result evidences the presence of closely located but differentially energized mitochondria, which allows them to also have different functional properties. Therefore, in many cell types, including skeletal muscle and adult cardiomyocytes, mitochondria exist as functionally and structurally distinct, non-connected organelles [3,9,16,46], which can be important for the specific cellular functions [21]. This may have important physiological consequences. For instance, depolarization and functional damage of distinct mitochondria under various pathological conditions will not result in a breakdown of the entire cell energetics. Moreover, a coupled mitochondrial network may provide a basis for Ca^2+^ tunneling (Ca^2+^ wave propagation), whereas mitochondrial discontinuity can prevent propagation of the Ca^2+^ signal and thus Ca^2+^ -mediated apoptosis.

Interestingly, imaging analysis provided evidence that cardiac mitochondria are firmly fixed and do not demonstrate electrical connectivity in adult rat cardiomyocytes (see above), but are subject to very low-amplitude fluctuations [16] probably due to changes in the assembly of the mitochondrial cristae and transitions of the inner mitochondrial membrane, observed previously by electron tomography microscopy and considered to reflect the metabolic state of mitochondria. In contrast, HL-1 cells with cardiac phenotype do not exhibit the strictly regular mitochondrial distribution typical for rat cardiac cells. In these cells, mitochondria can be highly dynamic and motile, undergoing continual fission and fusion [16,36]. It can be suggested that strictly different mitochondrial dynamics in adult cardiomyocytes and neonatal or HL-1 cells is responsible for their remarkably different connectivity and functional parameters.

### 2.4. Functional heterogeneity of mitochondria

A growing body of evidence has demonstrated that mitochondria not only vary between different types of cells and not only display dissimilar morphology, but that mitochondria localized in specific regions of a single cell may have different functional properties [3,5,6,10,13,14,45,52]. In contrast to biochemical determination, fluorescent confocal imaging affords the unique opportunity to directly visualize mitochondrial function separately in various mitochondrial subpopulations, small clusters or even in individual mitochondria [3,5,6,13]. Moreover, this technique is the direct approach to resolve the heterogeneity and the complex intracellular spatiotemporal organization of the mitochondria, mitochondrial signals and downstream events. It has been shown that mitochondria localized in different cell regions can have different morphology, biochemical properties and respiratory activities, demonstrating clear intrinsic heterogeneity [52]. Different mitochondrial subpopulations are present in cells (cf. Figure 2B,C) that may be differently involved in physiological [5] and pathological processes [53]. Imaging techniques have established the heterogeneity of mitochondrial redox potentials in skeletal muscle [5] and cardiomyocytes [10] and also with respect to mitochondrial reactive oxygen species (ROS) levels and uncoupling proteins (e.g. UCP-3) [6,46,54]. The heterogeneity of mitochondrial membrane potential, which reflects the functional status of mitochondria within cells, was detected in cultures of various cell types under normal growth conditions, cell stresses and apoptosis [3,6,12,13,45,55–57]. Notably, heterogeneous membrane potential has been shown even within individual mitochondrial filaments [58]. The existence of mitochondrial domains of heterogeneous electrical and redox potentials further demonstrates the existence of complex mitochondrial heterogeneity maintained by yet undiscovered processes. The heterogeneity of mitochondrial calcium has been demonstrated in cardiac cells [59]. Also, mitochondria may consist of subpopulations with differential sensitivity to calcium-induced inner membrane permeability transition, indicating that mitochondria are heterogeneous in their response to calcium [60].

Importantly, static heterogeneity of mitochondria can be distinguished from dynamic heterogeneity, where individual temporal behavior and dissimilar responses of mitochondria can be observed [13]. Distinct subsets of mitochondria can thus display different responses to substrates and inhibitors and vary in their sensitivity to pathology, apoptosis and oxidative stress [10,55,61]. For instance, in liver cells a very heterogeneous response to substrate addition was found [13]. It is known that mitochondrial flavoproteins are fluorescent in their oxidized state and that they initially show a relatively homogeneous redox state of mitochondria in hepatocytes. Strong reduction occurred in flavoproteins after adding substrate and resulted in a strong decline in fluorescence in almost all mitochondria. However, some small mitochondrial groups or individual mitochondria remained at almost the initial fluorescence, thus demonstrating their very different response to the substrate [13]. Authors suggested that this effect can not be explained by diffusion problems since “responding” and “non-responding” mitochondria were equally distributed in a hepatocyte irrespective of their distance from the cell membrane.

### 2.5. Possible physiological roles of distinct mitochondrial subpopulations

Previous findings suggest that the structural organization of the cell, as well as local energy and other cellular demands determine mitochondrial region-specific functional behavior, and lead to functional specialization of mitochondria [3,6]. Mitochondrial heterogeneity can be evident at various levels including specific cellular functions. Thus, different mitochondrial subpopulations, small clusters or even individual mitochondria may carry out diverse processes within a cell, depending on the particular cell-specific region and surrounding (Figure 2A). For example, the closeness of mitochondrial subpopulations to the plasma membrane (subsarcolemmal mitochondria, SS) (Figure 2B) may be important for functional coupling to ATP-driven ion pumps (e.g. Ca^2+^ entry) [62]. Moreover, this subpopulation may also defend intracellular structures against the high oxygen concentration outside the cell, serving as a “protective barrier” [6]. Mitochondria clusters surrounding the nuclei (Figure 2B) may serve to drive mitochondrial metabolism to generate ATP close to the nucleus as has been shown for parotid acinar cells [11]. This observation is in line with the concepts of an integrated phosphotransfer network and energetic channeling between mitochondria and nuclei suggested by Dzeja *et al.* [37]. Perinuclear subsets (PN) can therefore play an important physiological role in the mechanisms driving nuclear import, as well as in regulating a variety of other nuclear functions.

Monitoring of mitochondrial flavoprotein autofluorescence by confocal fluorescent imaging of intact or permeabilized skeletal muscle fibers (e.g. form M. soleus) indicated that different mitochondrial subpopulations may be highly heterogeneous with respect to their redox state (Figure 2C). In mouse skeletal muscle, a much more strongly oxidized state of subsarcolemmal mitochondria as compared with intermyofibrillar mitochondria has been demonstrated [5]. This problem was also addressed in the studies of muscle mitochondria by FACs analysis [5]. Using the confocal imaging technique, a similar phenomenon was also found in rat soleus (Figure 2C) and gastrocnemius muscles, where a higher oxidative state of mitochondrial flavoproteins also correlated with elevated levels of mitochondrial calcium as evidenced from the fluorescence signal of specific calcium probe Rhod-2 [6]. The metabolic differences between subsarcolemmal and intermyofibrillar mitochondria may have important functional and physiological consequences. Indeed, subsarcolemmal mitochondria are located close to the cell periphery and therefore exposed to higher oxygen levels than are other mitochondria inside the cell. Such localization close to the source of oxygen may explain the more oxidized state of this mitochondrial subset and potentially indicate a more active mitochondrial respiration. Subsarcolemmal mitochondria may serve as a protection barrier maintaining permissive levels of oxygen in the cell.

Therefore, this subsarcolemmal population of mitochondria may defend intracellular structures against the high oxygen concentration outside the cell and thus provide an important shielding mechanism against oxidative stress inside the cell. This is well consistent with the higher expression of uncoupling protein-3 (UCP-3) in subsarcolemmal mitochondria as compared with the intermyofibrillar subpopulation [54]. Increasing evidence indicates that mitochondrial uncoupling proteins (including UCP-3) play a central role in the regulation of mitochondrial ROS production by decreasing mitochondrial membrane potential (“mild uncoupling”) [63,64]. The formation of ROS is a function of surrounding oxygen concentration [65]. A higher content of UCP-3 may therefore compensate ROS overproduction as a result of the greater oxygen level near the cell membrane. On the other hand, a lower UCP-3 level may maintain more efficient ATP synthesis close to the myofibrils necessary for contraction. These data are in line with other reports, demonstrating that subsarcolemmal mitochondria exhibit a lesser degree of coupling and show significantly less oxidative damage, most probably due to a negative feedback mechanism with involvement of active UCP-3 [66]. The exposure to higher oxygen concentration also supports the role of the higher UCP content of this mitochondrial subpopulation as at least one important determinant of ROS regulation. Therefore, subsarcolemmal and intermyofibrillar mitochondrial subpopulations in skeletal muscles exhibit dissimilar bioenergetic properties (Figure 2C) and can be differently affected by physiological stimuli or differently involved in the mechanisms of cell stress and injury. In addition, subsarcolemmal and intermyofibrillar subpopulations are differently influenced by high-fat feeding [67].

### 2.6. Heterogeneity of mitochondrial ROS

Mitochondria are major producers of reactive oxygen species (ROS) and are also major targets for oxidative damage [68–70]. The increase in ROS concentrations above the level required for normal cell homeostasis causes damage of proteins, DNA, RNA, phospholipids and biological membranes. Intracellular overproduction of ROS has been proposed as mechanisms in ischemia-reperfusion injury, hypoxia-reoxygenation and various oxidative stress associated diseases.

On the other hand, a moderate formation of ROS as second messengers and signal molecules was shown to be crucial for diverse and important cellular signaling responses. Moreover, under specific conditions ROS may have protective effects, for example associated with mechanisms of myocardial preconditioning and mitochondrial uncoupling.

**Figure 3.**
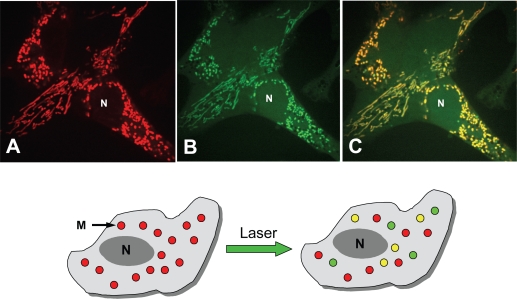
Heterogeneity of mitochondrial membrane potential and ROS during photooxidative stress in HL-1 cells revealed by confocal fluorescent imaging. *Upper panel:* Simultaneous confocal imaging of red fluorescence of mitochondrial membrane potential-sensitive probe TMRM, 0.1 μM (A), and green fluorescence of ROS-sensitive probe DCF, 20 μM (B). Fluorescence of TMRM and DCF is shown as a merge image (C). Some mitochondria with only green fluorescence indicate partially depolarized mitochondria with weaker TMRM signal and high levels of ROS production (ROS flashes). *Lower panel:* Red circles (M) show normally polarized mitochondria (membrane potential is monitored by TMRM fluorescence). Yellow circles show ROS-producing mitochondria (merged TMRM and DCF signal). Green circles are excessively ROS-producing and largely depolarized mitochondria, which demonstrate high ROS (high DCF green signal) and, in parallel, the loss of membrane potential (low TMRM red signal). N shows nucleus.

Heterogeneity of ROS was evident in various cells. Recent studies have demonstrated massive increases in localized ROS production during metabolic stress [10] and photostimulation. For instance, heterogeneous mitochondrial ROS production and large variations in mitochondrial membrane potentials were evident during photooxidative stress in adult rat cardiomyocytes [16,46], HL-1 and various carcinoma cells (MCF-7, HT-29) [6,13] (Figure 3). Live imaging analysis revealed that laser irradiation and laser-induced oxidative stress (Figure 3) can produce clear heterogeneity of the fluorescence of the mitochondrial membrane potential-sensitive probe TMRM (Figure 3A) and the ROS-sensitive probe DCF (Figure 3B), indicating heterogeneous depolarization of mitochondria due to local and heterogeneous ROS overproduction. The presence of different cellular sources for ROS localized in different cell regions (mitochondrial respiratory chain and various non-mitochondrial sources like plasma membrane NADPH oxidase, xanthine oxidoreductase, NO, etc.), their complex interactions (exchanges) and the phenomenon of “*ROS-induced ROS release*” [46,71] can be actively involved in the mechanisms of ROS heterogeneity and local ROS bursts. Very recently, using the mitochondrial matrix-targeted superoxide indicator, Wang *et al.* demonstrated the existence of superoxide flashes in individual mitochondria [51]. These ROS flashes could serve as a valuable biomarker for a wide variety of oxidative stress-related diseases. ROS can contribute to multiple essential intracellular signaling processes ranging from cell metabolism to ischemic preconditioning [72]. Also, various kinases and signaling cascades have been shown to be important in the mechanisms which may substantially modulate mitochondrial ROS, calcium and control mitochondrial induction of apoptosis. A role of the protein kinase C (PKC) family was recently demonstrated via pro-apoptotic protein p66Shc, which translates oxidative damage into cell death by acting as ROS producer [73]. Decreased mitochondrial ROS levels were also observed in the heart expressing the p38 MAPK activator MAPK kinase 6 (MKK6) [74]. Also, the tumor suppressor p53 can control ROS levels through its transcriptional target TIGAR [75]. Recently, a link between RAF survival kinase and changes in mitochondrial ROS and Ca^2+^, events preceding the onset of apoptotic cell death, was established, thus also indicating that signaling pathway, RAS-RAF-MEK-ERK cascade, AKT and Bcl-2 family proteins may actively participate in the regulation of mitochondrial ROS [33].

### 2.7. Heterogeneity of mitochondria in pathology

Mitochondria perform many important cellular functions (Figure 1A); on the other hand they themselves are very sensitive to energy and oxidative stresses. Heterogeneous mitochondrial damage is suggested in various pathologies including myopathies, ischemia reperfusion injury and apoptosis, where heterogeneous release of mitochondrial cytochrome *c* has been directly [76] and indirectly [77] demonstrated. Since mitochondrial damage inhibits oxidative phosphorylation and increases ROS generation, heterogeneity of injury would result in spatial heterogeneity of ATP and ROS levels. Both local energy deficit and elevated ROS generation are damaging for mitochondria of the particular cell region, causing, in turn, an increase in the extent of heterogeneity and thus demonstrating an amplification effect, in which excessive elevations in ROS or Ca^2+^ ultimately contribute to necrotic or apoptotic cell death. It can thus be expected that under various pathological conditions, such as metabolic diseases, cell stresses, ischemia reperfusion or under conditions of substrate deprivation, the extent of heterogeneity of mitochondrial function significantly increases, correlating with the degree or severity of injury that may have a critical impact on energy metabolism and cell viability.

The mitochondrial defects (defects in various complexes of the respiratory chain, e.g. COX deficiency) can be heterogeneously distributed in the tissue due to the phenomenon of their mosaic expression [78]. Distinct mitochondrial subsets may also vary in their sensitivity to pathology [6,10,53,55,56,79]. Mitochondrial defects can be heterogeneously distributed in various mitochondrial subpopulations like subsarcolemmal and intermyofibrillar mitochondria, which may be differently involved in pathological processes, such as ischemia reperfusion injury [53]. Interestingly, defects in mitochondrial dynamics may increase heterogeneity. For example, cells with targeted null mutations in fusion proteins Mfn1 or Mfn2 and lacking the ability for mitochondrial fusion show a high degree of mitochondrial functional heterogeneity [61].

Heterogeneity of mitochondrial redox state, membrane potential and calcium has been studied in cardiac cells under pathological conditions [55,59]. In permeabilized myocardial fibers, relatively homogeneous patterns of mitochondrial redox state, membrane potential and calcium have been documented by monitoring flavoprotein, TMRE and Rhod-2 fluorescence, also demonstrating a typical regular mitochondrial arrangement [9]. Under pathological conditions of ischemia reperfusion injury, however, myocardial cells showed irregularities in the fluorescence of mitochondrial flavoproteins due to regional differences in their redox state. Simultaneous imaging of Rhod-2 fluorescence indicated numerous discrete “black holes,” indicating mitochondria that lost calcium. Similar “black holes” in TMRE fluorescence detected many distinct depolarized mitochondria lacking TMRE sequestration due to the collapse of membrane potential [6,13]. These results show that ischemia reperfusion causes abnormal distribution of mitochondrial redox/electrical potentials and calcium because of heterogeneous mitochondrial damage in cardiac cells, which can be related to local ROS overproduction [77]. However, heterogeneous production of mitochondrial ROS is not only restricted to ischemia reperfusion, but indeed may be an important intermediate in intrinsic and extrinsic pathways of cell death.

Also, impairment of essential mitochondrial interactions and increased mitochondrial heterogeneity in the pathological state may lead to metabolic instability and consequently to severe cell and organ injury [71]. For instance, mitochondrial “ROS-induced ROS release” has been shown to result in the collapse of mitochondrial membrane potential and the destabilization of the action potential through a mechanism involving a mitochondrial inner membrane anion channel. This leads to the heterogeneity of mitochondrial membrane potential and ROS, contributing to abnormal electrical activation and arrhythmias in the whole heart during ischemia reperfusion [71]. The analysis of mitochondrial diversity, thus, may have important implications for the diagnosis of various mitochondria-related disorders, and the application of mitochondrial imaging opens a promising avenue for the development of a new diagnostic approach for detecting regional mitochondrial defects.

### 2.8. Heterogeneity of mitochondria in apoptosis

Mitochondria play a key role in inducing apoptosis by releasing the respiratory chain component cytochrome *c,* while, at the same time, mitochondrial function is inhibited by the loss of cytochrome *c*. On the other hand, the apoptosis machinery requires ATP produced by mitochondrial oxidative phosphorylation. Therefore, heterogeneous cytochrome *c* release would allow one mitochondrial population to be involved in apoptotic signaling, whereas another subset might be able to provide the ATP needed for apoptosis [80]. Such heterogeneity of cytochrome *c* release was indirectly demonstrated in the case of cardiac ischemia reperfusion injury [77]. Using the mitochondrial imaging approach, it was directly shown that ischemia reperfusion causes heterogeneous mitochondrial damage [55], which can be related to local ROS production and mitochondrial permeability transitions associated, in turn, with heterogeneous cytochrome *c* release (see above). Moreover, heterogeneous response of mitochondria during staurosporine (STS)-induced apoptosis has been shown at the single organelle level, where some mitochondria were depolarized while others were at their normal membrane potentials [57]. This unequal collapse of membrane potential thus demonstrated a phenomenon of mitochondrial heterogeneity that seems to be a supplementary event of the apoptotic process. Moreover, exploration of the mechanism behind heterogeneity revealed that BAD, a proapoptotic BCL-2 family member, can be considered a significant contributor influencing the level of mitochondrial heterogeneity [81].

### 2.9. Possible cellular mechanisms causing mitochondrial heterogeneity

The discovery of mitochondrial heterogeneity emphasizes the balance and possible relationship between various mitochondrial functions. Obviously, mitochondrial dynamics, including all processes of fusion-fission, mitochondrial motility and swelling-shrinkage may cause a variety of mitochondrial morphology in a cell. However, the origin and role of mitochondrial functional heterogeneity under physiological and pathophysiological conditions remain to be elucidated. Also, numerous questions remain unanswered, such as: 1) What are the intracellular mechanisms responsible for mitochondrial heterogeneity? 2) How do different metabolic and pathophysiological conditions, local concentrations of cytosolic factors and messengers, ROS, Ca^2+^ influence mitochondrial heterogeneity? 3) Is the heterogeneity of mitochondrial function defined genetically, or is it more dependent on environmental aspects? 4) What is the role of mitochondrial uncoupling proteins (UCPs) in local ROS regulations? 5) What is the role of cytoskeletal proteins here? 6) Very important question to answer in future research is also related to specific distribution and possible heterogeneity of proteins involved in mitochondrial dynamics (fission/fusion, motility, etc.) and/or binding of such proteins to a particular mitochondrial subpopulation.

**Figure 4.**
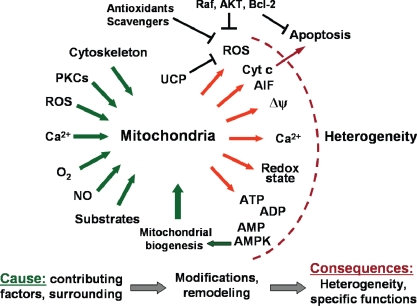
Scheme summarizing the specific questions and hypotheses regarding origin and possible mechanisms contributing to the heterogeneity of mitochondria and mitochondrial function.

Mitochondria are able to monitor their surrounding environment, including intracellular ATP levels, as well as ROS and Ca^2+^ [82] and the presence or absence of growth factors [32,33] (Figure 4). On the other hand, the existence of microdomains with restricted diffusion, functional enzyme coupling and channeling results in strong metabolic heterogeneity inside the cell. Therefore, the environment of each single mitochondrion can be significantly different from that of other mitochondria, potentially causing region-specific modifications of mitochondrial properties and function (Figure 4). Many intracellular factors like local Ca^2+^ levels, local ROS production, signaling proteins as well as oxygen and pH gradients may cause modification of mitochondria, leading to their heterogeneity [83,84]. Each of these factors could play different roles in ATP production and transfer to specific cellular regions, as well as in coordinating the functions of different mitochondrial subsets. Moreover, it can be suggested that the heterogeneity of mitochondria and mitochondrial function are dictated by the local ATP demand for specific cellular functions.

### 2.10. Various levels of mitochondrial heterogeneity

Various levels of mitochondrial heterogeneity can be considered including: *Heterogeneous morphology:* the presence of small spheres, short rod-like shape, spaghetti-like shape, long branched tubules, complex mitochondrial network. *Heterogeneous functional properties:* such as redox states, membrane potentials, respiratory activities, mitochondrial calcium and ROS levels, contents of uncoupling proteins (e.g. UCP-3), as well as phospholipid compositions of mitochondrial membranes. *Heterogeneous mitochondrial behavior:* like their different responses to substrates and inhibitors, different sensitivities to pathology, resistance to apoptosis, oxidative stress and starvation. *Heterogeneous dynamics:* including the simultaneous presence in some cells of different dynamic events of fusion-fission; small oscillatory movements in mitochondrial network; filament extension, retraction; fast and frequent oscillating branching; long-distance translocation of single mitochondrion or mitochondrial filament. Notably, fragmentation of the mitochondrial network may facilitate mitochondrial movement in cells, suggesting a link between mitochondrial motility and morphology.

## 3. Conclusions

Taken together, many previous findings suggest that the structural organization of the cell, as well as local energy (ATP) and other cellular demands (e.g. calcium-buffering capacities) and various requirements in mitochondrial signaling may determine mitochondrial region-specific functional behavior, leading to functional specialization of mitochondria. This suggests a strong relationship between mitochondrial function and cell organization, and the intracellular position of mitochondria may define their functional features. Thus, different mitochondrial subpopulations, clusters or even single mitochondria may carry out diverse processes within distinct regions of a cell (Figure 2A) and differently interact with other intracellular structures. Moreover, various functions of mitochondria can be associated with the features of their morphology and dynamics. The problem of heterogeneity points to a new level of mitochondrial complexity and can be important for understanding the basic mechanisms of mitochondrial function and regulation in normal cells and their dysfunction in various pathologies. The study of mitochondrial heterogeneity and the heterogeneity of mitochondrial function represents a new challenging direction in mitochondrial research, potentially integrating mitochondrial bioenergetics and cell physiology with various physiological and pathophysiological implications. Many aspects of mitochondrial heterogeneity certainly deserve further analysis including mathematical modeling and systems biology approaches in order to better understand mechanisms by which cells arrange specific interplay between various mitochondrial functions.
